# Geographical distribution of emergency obstetric and neonatal care signal functions in Ethiopian health facilities: 2021–2022 Ethiopian service Provision Assessment (SPA)

**DOI:** 10.1186/s12913-024-10893-5

**Published:** 2024-04-02

**Authors:** Dagne Deresa Dinagde, Habtamu Wana Wada

**Affiliations:** 1 Departments of Midwifery, College of Health Sciences, Mattu University, Mettu, Ethiopia; 2 Departments of Midwifery, College of Health Sciences, Arba Minch University, Arba, Minch Ethiopia

**Keywords:** BEmONC, CEmONC, EmONC, In ethiopia

## Abstract

**Background:**

The maternal mortality ratio in Ethiopia is still high, with an estimate of 412 deaths per 100,000 live births in 2016. Signal functions for emergency obstetric and neonatal care must be accessible and usable in order to successfully prevent maternal deaths. It is an important strategy to reduce maternal and newborn morbidity and mortality in countries with limited resources. Hence, an assessment of the availability of fully functioning EmONC services and their coverage per 500,000 people in Ethiopia is crucial.

**Methods:**

This study is a retrospective analysis of data from the Ethiopian Service Provision Assessment Survey (ESPA), a national-level survey data source. Data collection for the survey took place from August 11, 2021, to February 4, 2022. For this investigation, 905 healthcare facilities in total were evaluated for the availability of emergency obstetric and new-born care (EmONC) services at all hospitals, selected health centers, and private clinics were evaluated. Descriptive data analysis was done by the using statistical package for social science version 26 (SPSS) to run frequency and cross-tabs. Global Positioning System (GPS) (arc map 10.8) Software was used for spatial distribution in order to locate the physical accessibility of EmONC providing health facilities on flat map surfaces. It was projected based on Ethiopia’s geographic coordinate system at Adindan UTM zone 37^0^N.

**Results:**

Of 905 health facilities, only 442 (49%) could provide fully functioning BEmONC, and 250 (27.6%) health facilities have been providing fully functioning CEmONC. The overall coverage of BEmONC ratios in Ethiopia is 1.5–3.77 per 500,000 population and CEmONC (0.83–2.1) per 500,000 populations. Regions such as Amhara, SNNPR and Addis Ababa had found to have high BEmONC ratio. The geographical distribution of the EmONC showed that the central areas of the country, such as southwest Shewa and east Shewa, the Oromia region, the northern areas of the South Nation, nationalities, and peoples regions (SNNPR), including the Gurage zone and the Wolaita-Soddo zone, and the middle areas in the Amhara region (west Gojjam or around Bahir Dar town), and the southern areas, Debra Tabor and Debre Birhane zones, all had greater access to facilities offering complete EmONC services.

**Conclusion:**

Comprehensive emergency obstetrics and neonatal care (CEmONC) in Ethiopia met WHO recommendations, despite basic emergency obstetric and neonatal care (BEmONC) falling below those standards in Ethiopia. There are extremely large disparities in the accessibility of both basic and comprehensive emergency obstetrics and neonatal care in Ethiopia. Thus, Strategic planning is needed to improve infrastructures and inputs for EmONC services, particularly in remote areas of the country. Additionally, private facilities ought to place a priority on the provision of these services.

## Background

The ability to access emergency obstetric and newborn care (EmONC) services is a critical factor in determining the survival of both mothers and newborns after childbirth [1]. The vast health issue is maternal mortality, especially in sub-Saharan Africa, where more than half (70%) of all maternal fatalities globally take place [2]. Women who are experiencing obstetric-related difficulties can avoid death by receiving emergency obstetric and newborn care, which is a crucial sequence of life-saving procedures [3, 4]. Emergency obstetric and newborn care (BEmONC) is categorized as basic emergency obstetric and newborn care (BEmONC) and comprehensive emergency obstetric and newborn care (CEmONC). BEmONC can prevent up to 40% of intrapartum neonatal and maternal mortality [5].

BEmONC services include seven key activities: performing assisted vaginal deliveries, giving parenteral antibiotics, uterotonic medications, anti-convulsants, manual removal of the placenta, removing products of retained tissue, and performing neonatal resuscitation. CEmONC services require two extra signal functions in comparison to BEmONC services: transfusion of blood and cesarean Sect. [6].

According to the WHO report, most neonatal deaths are connected to intrapartum and immediate postnatal complications like asphyxia and the inability to breathe easily. The highest neonatal mortality ratio on the globe in 2020 will be found in Sub-Saharan Africa (27 deaths per 1000 live births), which accounts for 43% of all newborn deaths worldwide. Central and southern Asia are second (23 deaths per 1000 live births) [7].

Globally, more than half of maternal deaths (57%) occurred during the postpartum period, or after birth [8]. The majority of maternal deaths among postpartum mothers occur within 24 h of giving birth, followed by deaths within 7 days of delivery, which account for 50% and 20% of maternal mortality, respectively [9]. Most maternal deaths in Ethiopia occurred after delivery, accounting for an estimated 51–75% of all recorded maternal deaths. Most maternal deaths in Ethiopia occurred after delivery, accounting for an estimated 51–75% of all recorded maternal deaths. This is related to home delivery, facilities’ level barriers, and delays in treatments [10, 11].

Every day in Ethiopia, 15% of mothers need comprehensive emergency obstetric and newborn care (CEmONC) treatments because of serious obstetric issues [12]. Delay (lack of) in receiving appropriate and adequate treatments contributed more than one-third (36.3%), while lack of receiving medical treatment due to distance from health facilities contributed about 27% of social causes of maternal death in Ethiopia [13].The main causes of maternal deaths are due to direct obstetric complications: hemorrhage, infection, hypertension during pregnancy, prolonged or obstructed labor, and ruptured uterus, which account for about 80% of maternal deaths. This high rate of maternal mortality, especially in developing countries, is a reflection of unequal access to health services and emphasizes the wealth and poverty disparity [14].

To combat this, as part of the Sustainable Development Goal (SDG), the World Health Organization (WHO) aimed to implement various policies and programs to achieve a worldwide MMR lower than 70 per 100,000 live births by the end of 2030 [15]. In Ethiopia, EmONC was one of the strategies to achieve this goal, and it has a package of services necessary to manage the direct obstetric complications [16]. In order to prevent complications related to pregnancy during pregnancy, labor and delivery, and the postpartum period, a minimum of one CEmONC facility was intended to form five BEmONC facilities, which each service 500,000 mothers and their newborns [12].

Except for a few primary studies, no study has been found that shows the status of signal functioning for emergency obstetric and neonatal care among health facilities in Ethiopia. The present status of CEmONC and BEmONC services was provided in this study, which will also use GIS to pinpoint where these services are available in Ethiopia. To improve emergency obstetric and newborn care services in Ethiopia, this information is essential for planning, resource allocation, and human resource development.

## Methodology

### Study setting

Following the 2014 ESPA, the Ethiopia Service Provision Assessment (ESPA) for 2021–22 is the second survey of its sort. The public health care sector in Ethiopia is organized into a three-tier system of primary, secondary, and tertiary health care. The main Health Care Unit (PHCU) is made up of Tier one (main hospital, health center, and related satellite health posts). General hospitals make up Tier two, the secondary health care system, and specialty hospitals make up Tier three. With the aim of providing information on the general performance of facilities that provide maternal, child, and reproductive health services, services for specific infectious diseases, such as sexually transmitted infections (STIs), HIV/AIDS, tuberculosis (TB), and malaria, as well as the functions of the various components of the health system that may affect the quality of services, the survey was created to collect information from health facilities in Ethiopia.

### Study design and period

This study is a retrospective analysis of data from the Ethiopian Service Provision Assessment Survey (ESPA), a national-level survey data source. Data collection for the survey took place from August 11, 2021, to February 4, 2022, for the evaluation of all hospitals and sampled health centers, posts, and private clinics in Ethiopia.

### Eligibility criteria

All functioning hospitals in Ethiopia included in this study while, health posts and other health facilities that did not start operation were not evaluated for these services as they were not expected to provide them; they were excluded from this study. Additionally, because of the ongoing conflict between the federal government and the regional state administration during the data collection period, health facilities in the Tigray region were completely excluded from this study.

### Sample size determination

Based on the types of facility, all 413 hospitals in Ethiopia including 41 newly identified hospitals those governed by government and private institutions were included in the sample due to their modest number and significant contribution to the country’s healthcare system. A total of 310 health centers were obtained from the health centers. Due to their small number, all of the health centers in Dire Dawa and Harari were included in the sample. A sample of 356 clinics was taken in total. Due of their limited quantity, all of the higher clinics are represented in the sample. Due to the limited number of healthcare facilities in the Harari region, all clinics are from the region included. Out of 1407 health facilities, 249 were permanently closed, unreachable, and under security concerns and transformed into COVID-19 centers. 253 health facilities canceled for analysis due to having missing of full information (193) and incomplete infrastructure to provide EmONC services (60). Thus, finally this study conducted on 905 health facilities in Ethiopia.

### Sampling procedures

**Fig. 1:**
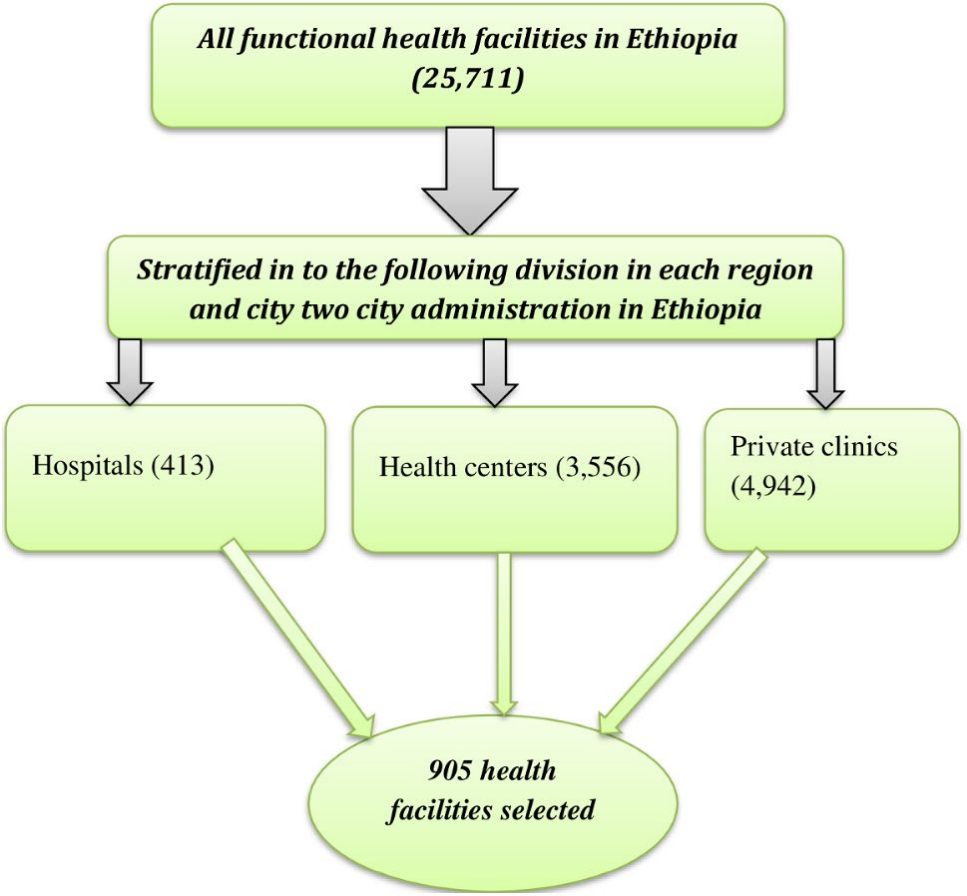
schematic presentation of sampling procedures among health facilities in Ethiopia, SPA 2021/22

A master list of 25,711 operational health facilities in Ethiopia was received from the Ministry of Health, excluding the Tigray area. 905 facilities in all were chosen, including 32 referral hospitals, 124 general hospitals, 218 primary hospitals, 310 health centers, 270 health posts, 19 specialty/higher clinics, 139 medium clinics, and 103 lower clinics, for this survey. The ESPA sample for 2021–2022 is a stratified random sample of 905 healthcare facilities that was chosen using sampling allocation procedures and equal probability systematic sampling. In order to achieve stratification, each region’s healthcare facilities were first divided into several types of facilities. The clinic category (higher, medium, lower, or specialized clinics) was then used to further stratify all of the clinics in each region [17] (Fig. 1). This is because all these facilities are quite different on services they are providing and were considered as heterogeneous. For instance, Hospitals and health centers often differ in terms of the range and complexity of services they provide, as well as the resources available to them. Stratifying them separately can help capture these differences and enable more nuanced analysis [18].

### Study variables

Outcome variables: To assess the *availability of EmONC services (Yes/No)* and its distribution among selected Ethiopian health facilities. BEmONC and CEmONC were both included in EmONC services. BEmONC services were supposed to be offered by all healthcare facilities, but CEmONC services were limited to hospitals in Ethiopia.

### Operational definitions

#### Availability of CEmONC/ BEmONC services

BEmONC performs seven major activities: performing assisted vaginal deliveries, giving parenteral antibiotics, uterotonic medications, anti-convulsants, manual removal of the placenta, removing products of retained tissue, and performing neonatal resuscitation. While the CEmONC service performs life-saving procedures such as surgery (cesarean section) and blood transfusions, in addition to activities to be performed by BEmONC. For every 500,000 people, there should be five facilities that can provide completely operational BEmONC and one site that can provide fully operational CEmONC [19].

#### Fully functioning EmONC facilities

All necessary signal functions included under EmONC are provided by these facilities [19, 20].

#### Partially functioning EmONC facilities

EmONC facilities that lack one or more signal functions are said to be partially functional. Hospitals that had completed seven or eight signal functions were categorized as CEmONC facilities that were only partially functional. Health facilities that offered five or six signal functions were likewise categorized as BEmONC facilities that were only partially operational. Non-EmONC facilities were those that did not meet these requirements [17, 20].

#### Data collection tool

Information about the facility’s preparedness to offer each of the priority services was gathered using the facility inventory questionnaire. In addition to gathering data on the location and operational state of the facility, the facility inventory questionnaire also gathers details on the availability of specific items, facility infrastructure, including the service delivery environment, and support system components like logistics, maintenance, and management. The inventory questionnaire was divided into three modules: module one, which asked about service availability; module two, which asked about general facility readiness; and module three, which asked about service-specific readiness [17].

#### Data quality management

Following the completion of the final ESPA questionnaires in English, translations into Amharic and Afan Oromo were created. Subsequently, the facility inventory questionnaires in English, Amharic, and Afan Oromo were loaded into tablet computers, which were used to record responses and provide questions during interviews. The responses to the paper survey questionnaires were input into computers and modified in the field using a method known as computer-assisted field editing (CAFE). The questionnaires were pretested to detect possible problems in the flow of the questionnaires, to determine how long it will take to conduct the interviews and to find translation problems. Additionally, the pretest assisted in identifying issues with the data entry scripts created for the CAFE activities. Then, From July 7 to August 4, 2021, thirty-seven team leaders and 148 interviewers, mostly health providers (nurses, nurse midwives, and clinicians) employed by EPHI were trained as interviewers in the use of the questionnaires and computer programs. Six regional coordinators, two data managers, and two central coordinators were among the 23 master trainers who led the main training for the 2021–22 ESPA.

#### Data processing and analysis

Data was taken from the SPA dataset and cleaned by running frequency using SPSS version 26 to check the absence of missing values and internal consistency. Then, descriptive analysis was performed using SPSS. The availability of EmONC was tested using descriptive statistical methods after variables had been recoded, re-categorized, and defined. Tables for category variables, frequency, and percentages were used to summarize each descriptive statistic.

#### Spatial data analysis

In this study, spatial autocorrelation and hot spot analysis were performed using Arc Map V.10.8 software [21]. To determine if EmONC services were randomly distributed, clustered, or scattered across space, the global spatial autocorrelation statistic (Global Moran’s I) was used. The distribution of EmONC services is dispersed, clustered, and random, respectively, according to Moran’s I value, which is near to -1, + 1, and 0 in Ethiopia. To determine whether the EmONC services is a hot or cold spot, a hot spot analysis (Getis-Ord Gi*) was conducted. Z-scores and p values were used to calculate the hot and cold spot values for spatial clusters. In addition, the spatial interpolation analysis, which makes use of Kriging ordinary interpolation, was utilized to predict the level of EmONC services for values that were not sampled or measured from sampled data.

#### Intra-cluster correlation

The STATA post-estimation command “estat icc” was used to determine the intra-class correlation (ICC), which displays the percentage of between-cluster variation in the overall variation [22].

## Results

### Characteristics of health facilities

In total, 905 healthcare facilities nationwide, 374 hospitals, 270 health centers that were randomly chosen and 261 clinics were included in the findings about the availability of signal functions for emergency obstetric and neonatal care. The majority (63.5%) of the healthcare facilities included in this analysis were public, while 33.6% were for-profit private hospitals. The smallest number of health facilities (37) were surveyed in Benishangul Gumuz regional state, and the highest number (199) were in Oromia regional state. Almost all (91%) of these facilities are fully functional, and no more are under construction or expansion (Table 1).

**Table 1:** Characteristics of health facilities assessed by type of facilities (*N* = 905)

Facility type	Managing authority				
	Public	Other governmental	Private for profit	NGO	Total
Referral Hospital	30	1	1	0	32
General Hospital	37	1	46	4	124
Primary Hospital	192	4	17	5	218
Health Center	266	0	0	4	270
Higher Clinic	0	0	15	0	15
Medium Clinic	4	1	124	10	139
Lower Clinic	1	2	98	2	103
Specialty Clinic	0	0	3	1	4

### Functional status of the facilities

As operationalized above, for the facility to be considered to have provided high-quality BEmONC services over the three months prior to the survey, seven signal functionalities had to be provided. A facility had to have carried out all nine signal functions in order to meet the standards for the CEmONC certification.

**Fig. 2:**
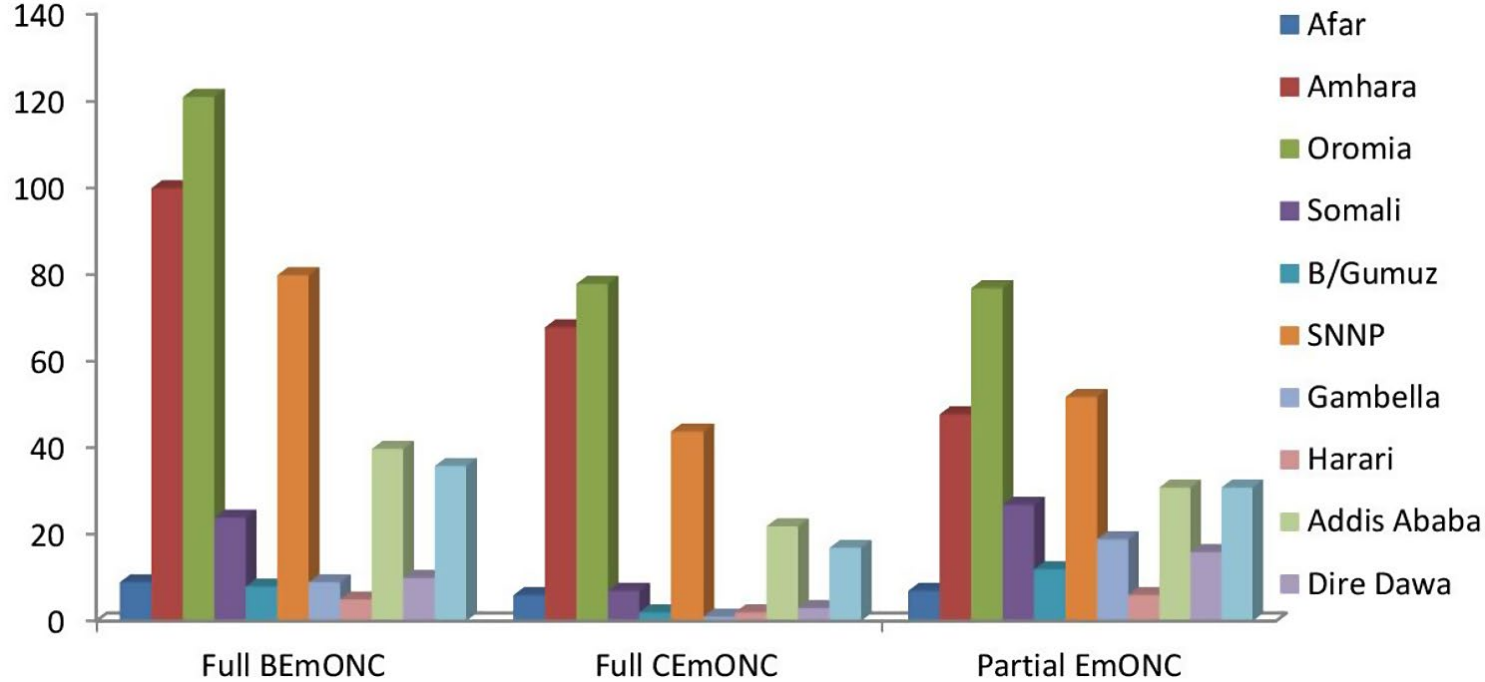
functional status of signal EmONC in Ethiopia by region, SPA 2021/22

**Fig. 3:**
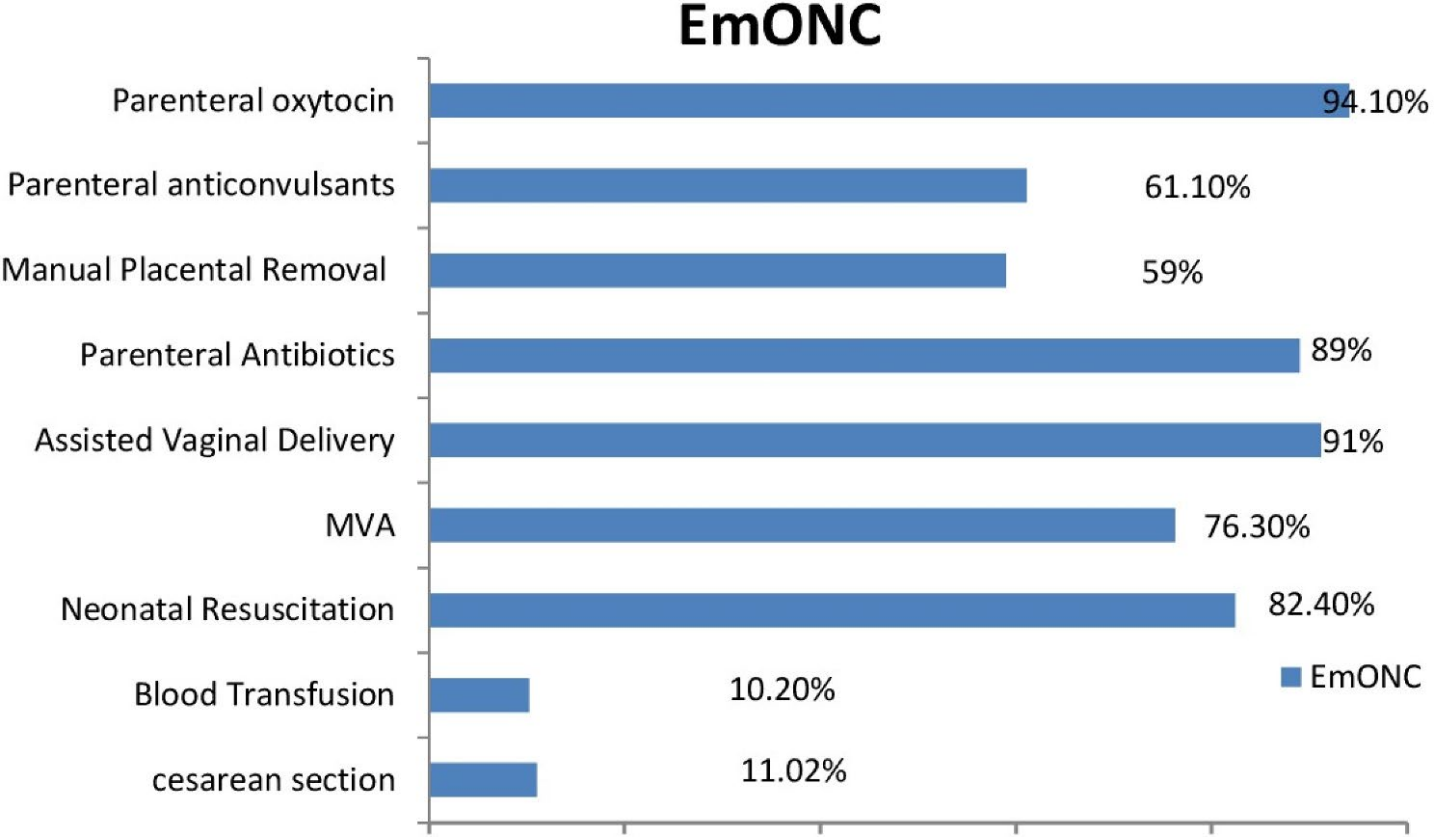
the nine key activities in emergency obstetric and new born care, SPA 2021/22

In general (Fig. 2), about half (49%) of all health facilities could provide fully functioning BEmONC, and only 27.6% of all health facilities could provide fully functioning CEmONC. When compared to other locations in Ethiopia, it was discovered that the Oromia regional state offers the highest accessibility to completely functional EmONC, with the Gambella region offering modest signal-operating EmONC. Parenteral oxytocin administration (94%) and assisted vaginal delivery (AVB) (91%) are the signal functions carried out most frequently over the past three months, whereas blood transfusions, which were carried out in 10% of facilities, were the least common BEmONC. parenteral anticonvulsants (89%) More than eight out of ten healthcare facilities can perform neonatal resuscitation, and six out of ten facilities administer parenteral oxytocin and perform manual removal of the placenta, while 68% of facilities can do neonatal resuscitation. More than half of facilities manually removed residual products of conception (MVA) and the placenta. Just nearly one in ten facilities performed a Caesarean delivery (Fig. 3).

Generally, compared to other facility types, hospitals are more likely to offer fully functional emergency obstetric and neonatal care (EmONC), and government facilities are more likely to do so than other managing authorities. In comparison to other regions, the signal function of EmONC is less likely to be available in the Gambella and Harari regions

### Overall coverage of fully functioning EmONC

The coverage of fully functioning BEmONC/CEmONC is calculated using the number of EmONC facilities per 500,000 people, as done below [14, 19, 20].

- Ethiopia population in the year of data collection = 123 × 10^6^ [23].
- Number of existing fully functioning BEmONC = 442.
- Number of existing fully functioning CEmONC = 250.

$${\rm{Rate}}\,{\rm{of}}\,{\rm{BEmONC}}\,{\rm{per}}\,{\rm{500,000}}\,{\rm{Population}}\,{\rm{ = }}\,{{{\rm{Existing}}\,{\rm{BEmONC}}} \over {{\rm{Total}}\,{\rm{Population}}}}\,{\rm{X}}\,{\rm{500,000}}$$

$${\rm{Rate}}\,{\rm{of}}\,{\rm{CEmONC}}\,{\rm{per}}\,{\rm{500,000}}\,{\rm{Population}}\,{\rm{ = }}\,{{{\rm{Existing}}\,{\rm{CEmONC}}} \over {{\rm{Total}}\,{\rm{Population}}}}\,{\rm{X}}\,{\rm{500,000}}$$

- In Ethiopia.
- Thus, ratio of BEmONC per 500,000 people = 1.8.
- Ratio of CEmONC per 500,000 people = 1.02.
- Regions.
- Region with the highest and lowest ratio of BEmONC respectively = Addis Ababa city (3.77) and Oromia region (1.5).
- Region with the highest and lowest ratio of CEmONC respectively = Addis Ababa city (2.1) and B/Gumuz (0.83).

There were roughly 123 million people living in Ethiopia at the time of the study. Based on the performance of the WHO EmONC guidelines signal functions, just 442 and 250 of the randomly chosen medical facilities had been offering BEmONC and CEmONC services, respectively. Accordingly, it was found that there were huge differences between BEmONC coverage (3.77 in Addis Ababa per 500, 000 populations) and the lowest in the Oromia region, while CEmONC coverage was 2.1 in Addis Ababa and 0.83 in Benishangul Gumuz (Table 2).

**Table 2:** shows the coverage of BEmONC / CEmONC in health facilities of each region (*N* = 905)

**Regions**	**BEmONC coverage**	**CEmONC coverage**
Afar	2.1	0.9
Amhara	3.45	1.5
Oromia	1.5	1.8
Somali	1.7	1.2
B/Gumuz	1.9	0.83
SNNPR	3.2	1.43
Gambella	2.6	1.2
Harari	1.7	1.6
Addis Ababa	3.77	2.1
Dire Dawa	2.31	1.8

### Availability of trained staffs

**Fig. 4:**
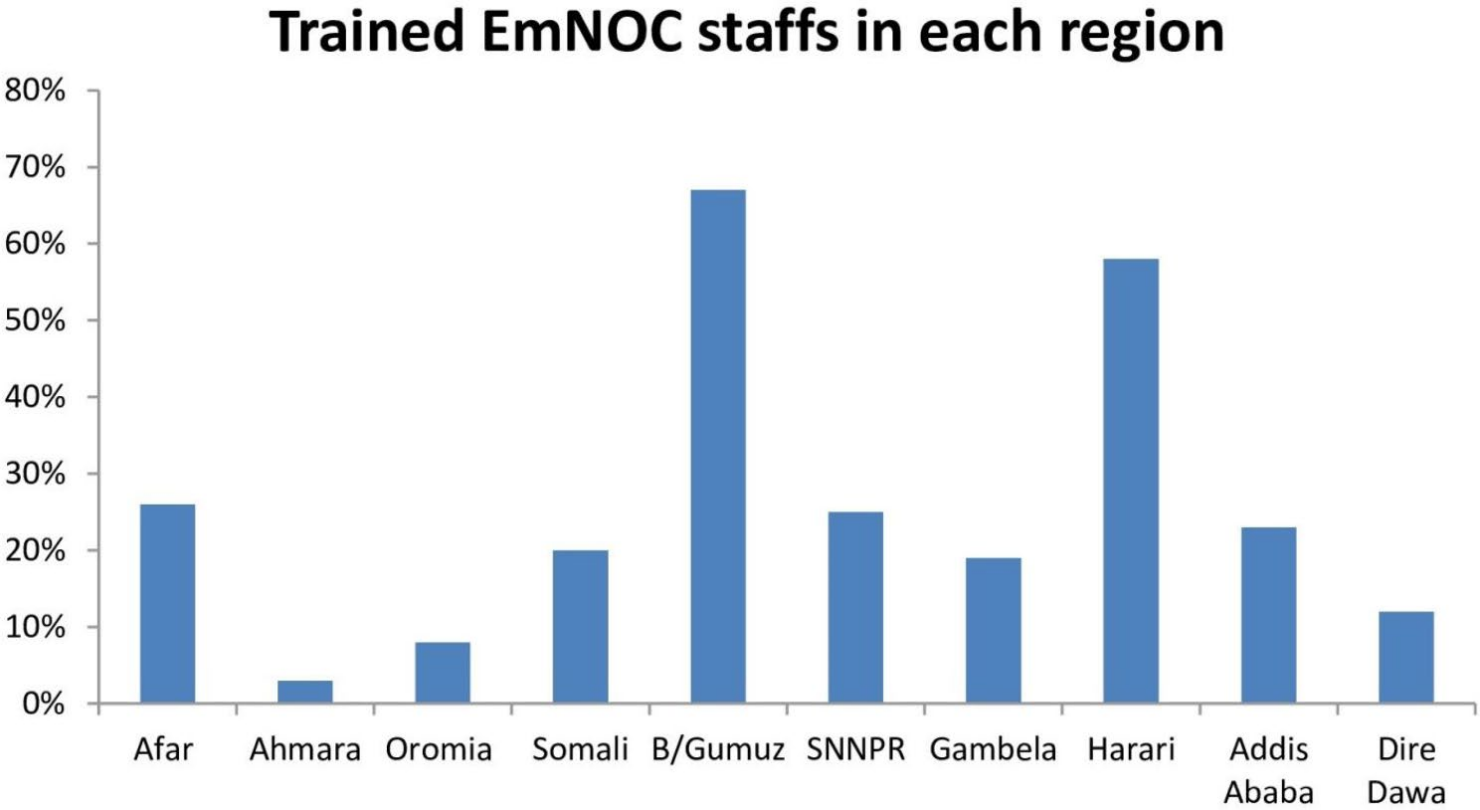
staffs trained in emergency obstetric and new born care, SPA 2021/22

Compared to the other facility types, a higher percentage of providers (63%) worked in general hospitals. The Amhara region had the lowest percentage of skilled providers in BEmONC and CEmONC, only 3%. While the Benishangul Gumuz region and the Harari region had the greatest percentage of 67% and 58% trained staffs, respectively (Fig. 4).

### Spatial distribution of EmONC in ethiopia

**Fig. 5:**
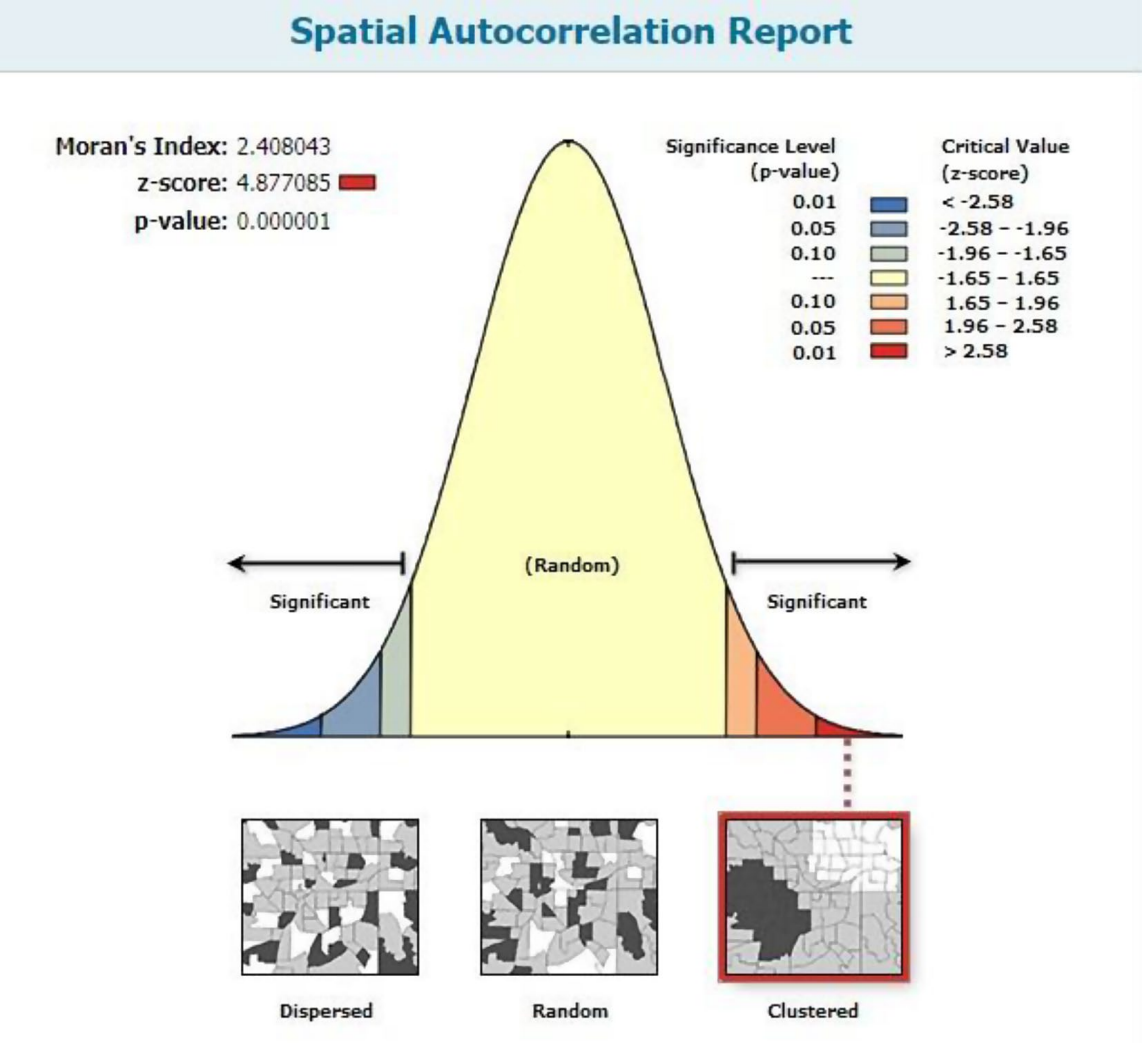
Spatial autocorrelation report of EmONC in Ethiopia, SPA 2021/22. Given the z-score of 4.87708510655, there is a less than 1% likelihood that this clustered pattern could be the result of random chance

The spatial autocorrelation is measured by the Moran’s I index, which makes it possible to determine whether or not the EmONC in a certain cluster is comparable to that of nearby zones. A higher level of significance is indicated by the vivid red and blue hues (to the end of the tails). It tests the hypothesis that there is no spatial autocorrelation for the response variable or assesses if the stated pattern is clustered, scattered, or random. In this investigation, EmONC’s estimated Global Moran’s Index value was 2.40. Apart from examining Moran’s I to determine the presence of a geographic correlation, the P-value (0.0001) was discovered to be below 0.05, indicating noteworthy proof of spatial autocorrelation in EmONC (Fig. 5).

### Cluster and outlier analysis of EmONC

**Fig. 6:**
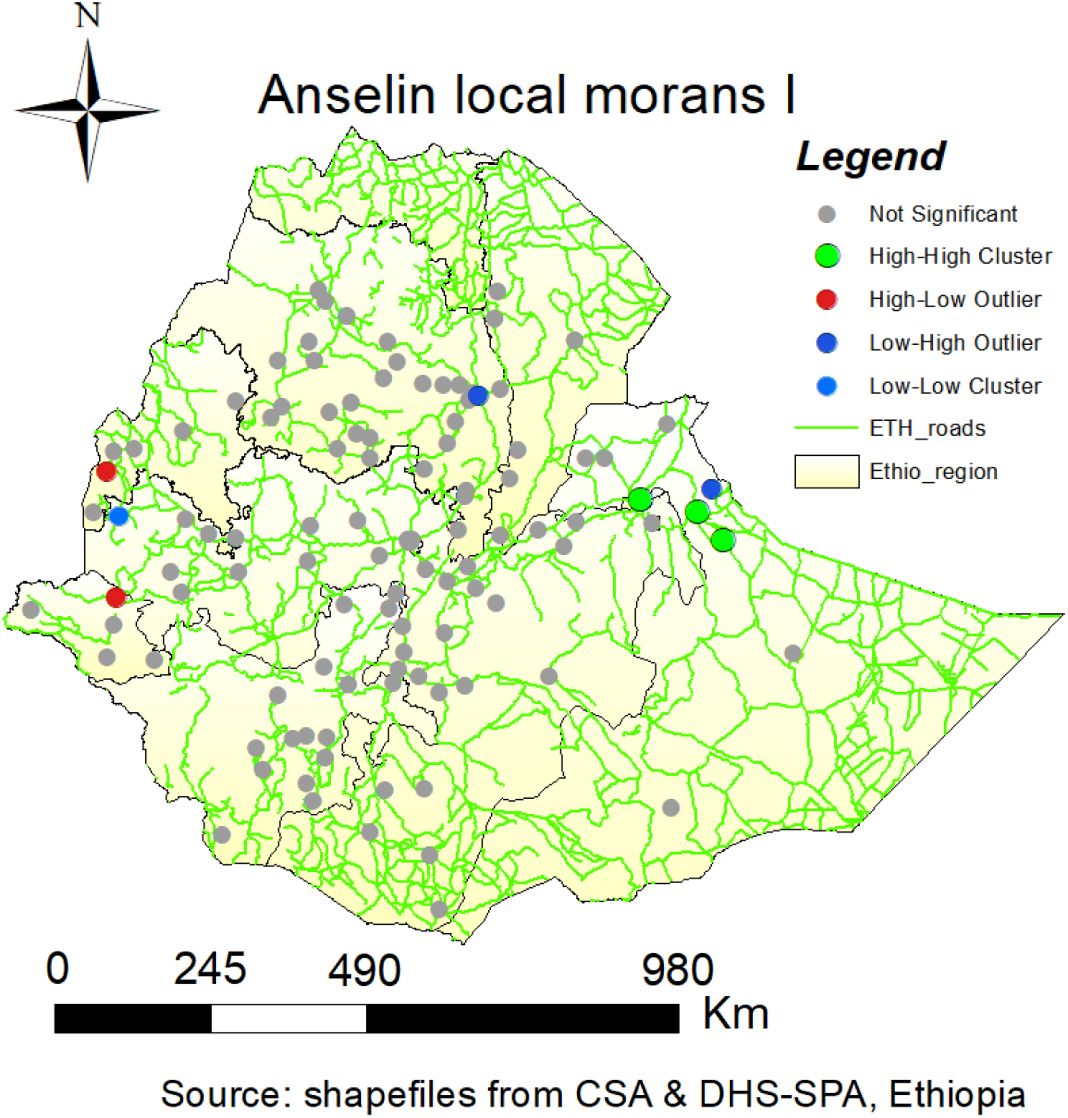
Cluster and outliers analysis for delayed age marriage in Ethiopia, SPA 2021/22

Using Anselin, a cluster and outlier analysis was performed to determine the type of grouping. Regional Moran’s I. An elevated positive Local Moran’s I value pointed out that if a feature is a component of a cluster, it means that it contains nearby features with equally high or low attribute values. A low local Moran’s I value shows that a feature has nearby features with different values. This characteristic is an outlier, and in order for the cluster or outlier to be deemed statistically significant, the P-value needs to be low. In the Somali region, areas around Jijiga, Fafan Zone, and Dire Dawa Administration City are some of the areas that have high clustering of the EmONC (Fig. 6).

### Hotspot analysis of EmONC services in ethiopia

**Fig. 7:**
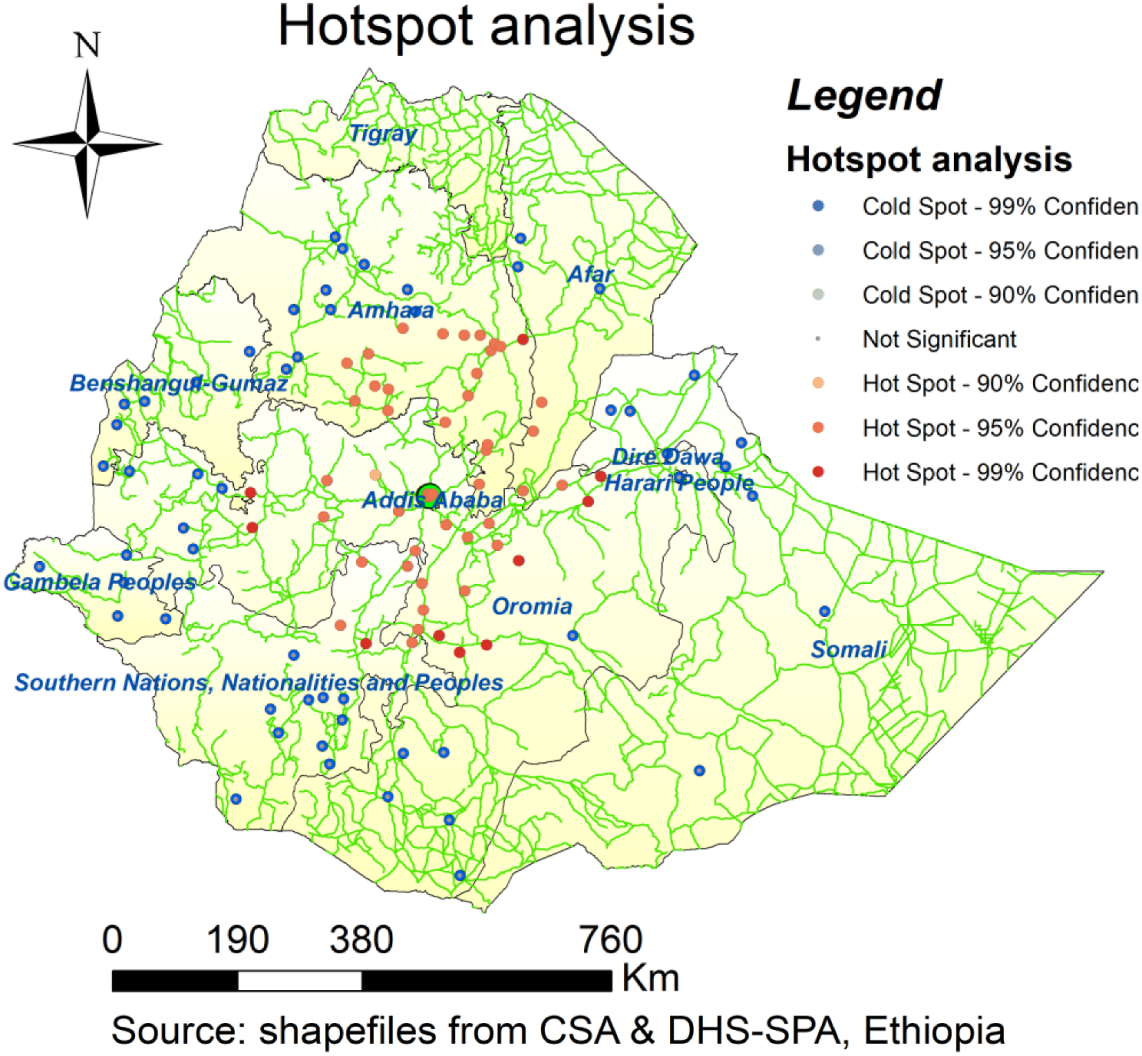
Hotspot analysis of fully functioning signal of EmONC, SPA 2021/22

In Ethiopia, (Fig. 7) shows regions with access to health facilities that offer fully working EmONC in the three months before the survey. The central areas of the country, such as southwest Shewa and east Shewa, the Oromia region, the northern areas of the South Nation, nationalities, and peoples regions (SNNPR), including the Gurage zone and the Wolaita-Soddo zone, and the middle areas in the Amhara region (west Gojjam or around Bahir Dar town), and the southern areas, Debra Tabor and Debre Birhane zones, all had greater access to facilities offering complete EmONC services. While outlying areas (regions far from the center and close to borders), such as Somalia, southern Oromia, the northernmost tip of Amhara, and Afar, had less accessibility to functional EmONC. Only around one-fifth of the facilities lacked access to a road for automobiles.

### Interpolation of EmONC/spatial prediction of EmONC

**Fig. 8:**
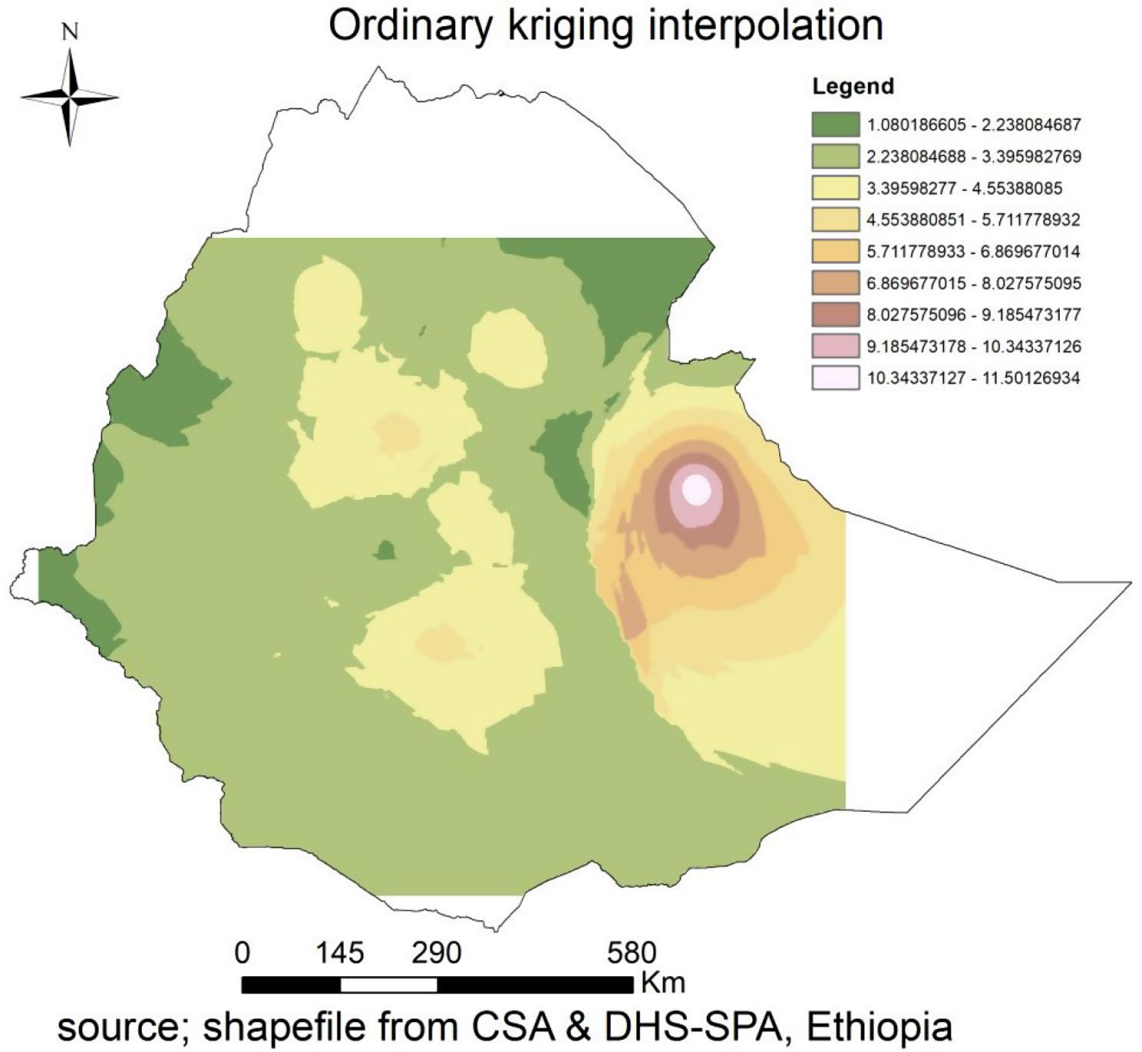
Ordinary kriging interpolation of EmONC services in Ethiopia, SPA 2021/2022

According to the ordinary Kriging interpolation of EmONC, the void demonstrated the locations that were not sampled. The whitish color predicts high possibilities for areas of EmONC services. Thus, the areas near Jijiga tend to have a high prediction of EmONC, while the Afar region and the western corner areas of the country (black green) have a low prediction of EmONC (Fig. 8).

#### Variation due to clusters

The null model’s ICC (the model that just includes the outcome variable) was 0.384, indicating that around 38.4% of the EmONC services overall variation was due to differences between clusters.

## Discussion

This study has demonstrated the Geographical distribution of Emergency Obstetric and Neonatal Care Signal Functions in Ethiopian Health Facilities from SPA. According to this study, about half of health facilities in Ethiopia have been providing fully functioning EmONC services, and the overall coverage of facilities with full signal function for basic emergency obstetric and neonatal care (BEmONC) was 1.8 per 500,000 population, which is below the WHO recommendation, while the overall coverage of CEmONC was 1 per 500,000 population, which was in line with the WHO recommendation. According to International Standards (WHO), at least five obstetric and newborn care facilities per 500,000 populations are needed [15].

This study is in line with research done in Ethiopia, Tanzania, and Cameron that discovered that EmONC availability is below the WHO recommendation. According to the Tanzanian study, 1 CEmONC facility and 2 BEmONC facilities were needed for a total population of 214,454. Inadequate EmONC facilities were found in the Cameroon study (2.2 BEmONC facilities per 500,000 people and 0.6 CEmONC facilities per 500,000 people) [24–26].

However, this finding is lower than the studies conducted in southwestern Ethiopia, with an overall BEmONC coverage of 5 per 500,000 people [19], Tanzania, which had an overall coverage of 8 per 500,000 people [27], and Bangladesh, which had 9 in 10 facilities providing full functional EmONC. Demographic differences may be the reason for this significant variation.

Based on the performance of the WHO EmONC guidelines signal functions, just 442 and 250 of the randomly chosen medical facilities had been offering BEmONC and CEmONC services, respectively in Ethiopia. Accordingly, it was found that there were huge differences between BEmONC coverage (3.77 in Addis Ababa per 500, 000 populations) and the lowest in the Oromia region, while CEmONC coverage was 2.1 per 500, 000 populations in Addis Ababa and 0.83 per 500, 000 populations in Benishangul Gumuz per 500, 000 populations. This might be due to, the noteworthy and favorable coverage of EmONC services was situated in a substantial and well-established urban area. This is brought on by a high population density. Thus, there is high demand for more EmONC services.

The spatial distribution of EmONC is not random and there is variation across Ethiopia. The central areas of the country, such as southwest Shewa and east Shewa, the Oromia region, the northern areas of the South Nation, nationalities, and peoples regions (SNNPR), including the Gurage zone and the Wolaita-Soddo zone, and the middle areas in the Amhara region (west Gojjam or around Bahir Dar town), and the southern areas, Debra Tabor and Debre Birhane zones, all had greater access to facilities offering complete EmONC services. While, outlying areas (regions far from the center and close to borders), such as Somalia, southern Oromia, the northernmost tip of Amhara, and Afar, had less accessibility to functional EmONC. This finding is similar to a study conducted in Ethiopia [21]. This may be justified by the fact that a high population density is observed in the country’s center due to urbanization, which calls for adequate healthcare facilities, as well as the fact that many businesspeople and investors who invest in the expansion of hospitals and health centers opt for the region because it is strategically located close to the capital city and has good access to roads.

Blood transfusion and cesarean section were the least-available services among all nine signal functions in both the public and private sectors, but the maternal and neonatal life-saving procedure was only available in 10 and 11% of health facilities. On the other hand, blood transfusion and cesarean section services were available only in 10% and 11% of Ethiopian health facilities, respectively. By narrowing the scope, of all the selected hospitals expected to provide blood transfusion and cesarean section services, only 60% and 81.3% could provide the service, respectively. Almost all, or 99%, of cesarean sections and blood transfusions are provided in public health facilities in Ethiopia. Additionally, nine out of ten facilities providing EmONC services were managed by Ethiopian governments. This is because public facilities offer all of these services for free. This finding is in contrast with a study conducted in Bangladesh, where most cesarean sections were conducted in private clinics [28]. However, this finding was in contrast to study conducted in Nigeria [29] which pointed out, A few individuals expressed their distrust towards healthcare facilities owned by the government. The doubt was expressed about how these clinics handled outpatients and performed cesarean sections without indication.

Finally, this study revealed that there is a significant inter-regional variation in EmONC coverage. As a result, Oromia and Benishangul Gumuz areas had the lowest signal function coverage of EmONC services, while Addis Ababa city accounts for the best overall coverage of EmONC. Studies conducted in Tanzania, India, Ghana, and Argentina validated this conclusion [27].

In terms of trained human power on EmONC services; The Amhara region had the lowest percentage of skilled providers in BEmONC and CEmONC, only 3%. While the Benishangul Gumuz region and the Harari region had the greatest percentage of 67% and 58% trained staffs, respectively. This could be as a result of the Ethiopian government and non-governmental organizations placing a great deal of focus on rural areas where maternal and newborn deaths could occur.

## Conclusion and recommendation

This study has shown that although CEmONC facility ratio complied with the basic requirements of international standards, BEmONC facility ratio falls short of the minimum required for Ethiopia’s population size.

### Recommendation for policy makers

Strategic planning is needed to improve infrastructures and inputs for EmONC services, particularly in remote areas of the country. The upgrade of health centers to full BEmONC providers, hospitals to full CEmONC and BEmONC providers, and the concurrent hiring of more EmONC provider personnel are crucial.

### For private hospitals and clinics

EmONC services should be prioritized by private clinics since they minimize maternal mortality by 75% when obstetric complications are appropriately managed.

## Data Availability

The data used for the analysis of the present study is available at https://dhsprogram.com/data/dataset/Ethiopia_Standard-DHS SPA 2021/2022.cfm.

## Abbreviations

BEmONC: Basic emergency obstetric and neonatal care
CEmONC: Comprehensive emergency obstetric and neonatal care
CAFÉ: Computer-Assisted Field Editing
EmONC: Emergency obstetric and neonatal care
EPHI: Ethiopian Public Health Institute
MMR: Maternal Mortality Rate
SPA: Service Provision Assessment
SNNPR: South Nations, Nationality and peoples of Region
WHO: World Health Organization
